# Reappraisal of Oral Steroid Therapy for Myasthenia Gravis

**DOI:** 10.3389/fneur.2020.00868

**Published:** 2020-08-25

**Authors:** Tomihiro Imai, Shigeaki Suzuki, Yuriko Nagane, Akiyuki Uzawa, Hiroyuki Murai, Kimiaki Utsugisawa

**Affiliations:** ^1^Department of Neurology, Sapporo Medical University Hospital, Sapporo, Japan; ^2^Department of Neurology, Keio University School of Medicine, Tokyo, Japan; ^3^Department of Neurology, Hanamaki General Hospital, Hanamaki, Japan; ^4^Department of Neurology, Graduate School of Medicine, Chiba University, Chiba, Japan; ^5^Department of Neurology, International University of Health and Welfare, Narita, Japan

**Keywords:** myasthenia gravis, oral corticosteroids, treatment strategies, cross-sectional study, logistic regression analysis

## Abstract

Treatment with oral corticosteroids at high doses with an escalation and de-escalation schedule is effective against myasthena gravis (MG). In fact, the use of corticosteroids has led to a reduction in mortality to below 10% after the 1960s. However, long-term use of oral steroids above a certain dosage level is known to cause a number of problems. In 2014, the Japanese clinical guidelines for MG proposed that the first goal in MG treatment (treatment target) should be set at minimal manifestations (MM) with oral prednisolone (PSL) 5 mg/day or below, and that treatment strategies should strive to attain this level as rapidly as possible. In 2015, a multicenter, cross-sectional study revealed that higher PSL dose and longer PSL treatment do not ensure better outcome. In the absence of good response, the PSL dose should be decreased by combining with modalities such as plasma exchange/plasmapheresis and intravenous immunoglobulin (fast-acting treatments). In 2018, we conducted a multicenter, cross-sectional study in a large population of Japanese patients with generalized MG, aiming to elucidate the correlation between oral PSL regimens and achievement of treatment goals. The ORs for low vs. high dose to achieve treatment goals at 1, 2, and 3 years were 10.4, 2.75, and 1.86, respectively, whereas the corresponding ORs for low vs. medium dose were 13.4, 3.99, and 4.92. Early combination with fast-acting therapy (OR 2.19 at 2 years, 2.11 at 3 years) or combination with calcineurin inhibitors (OR 2.09 at 2 years, 2.36 at 3 years) were also positively associated with achieving treatment goals. These results indicate that early combination of low-dose PSL regimens with other therapies is the key for early achievement of treatment goals in generalized MG. However, even with this regimen, ~35% of patients did not achieve the treatment target after 3 years. These results suggest the limitation of the current oral corticosteroid therapy. We need to develop new treatment options to increase the rate of satisfactory outcome.

## Introduction

Oral corticosteroids remain the primary treatment for generalized myasthenia gravis (MG), although various other disease-modifying therapies have emerged (1). Primary disease-modifying therapies for MG include immunosuppression therapy using oral prednisolone (PSL), azathioprine, cyclosporine, mycophenolate mofetil, and tacrolimus (2–6). Methotrexate, another immunosuppressant, is an effective steroid-sparing agent having similar efficacy and tolerability to azathioprine (7). On the other hand, additional immunomodulatory therapies may be required for aggressive exacerbations of MG, such as plasma exchange/plasmapheresis (PE/PP) and intravenous immunoglobulin (IVIg) (8–13). For patients receiving low-dose prednisolone, treatment goal is usually set at minimal symptoms (MM) according to the Myasthenia Gravis Foundation of America (MGFA) postintervention status (14). To achieve the treatment goal, various immunosuppressive agents have been added to corticosteroids as steroid-sparing agents at the start of treatment (5, 15–18).

This short review will provide an overview of corticosteroid treatment for generalized MG, and introduce a favorable regimen of oral corticosteroids for generalized MG based on a nationwide survey in Japan.

## History of Corticosteroid Treatment for MG

In 1935, Simon (19) reported the effects of treating MG with anterior pituitary extract. This was probably the first description of the therapeutic effect of corticosteroid-related agents on MG. Subsequently, many reports of small-scale studies in the 1950s and 1960s described favorable effects of adrenocorticotropic hormone and corticosteroids on MG. Grob et al. (20) underscored the fact that the use of corticosteroids led to a reduction in mortality to below 10% after 1966.

Prednisone and prednisolone are the oral corticosteroids commonly used for MG treatment. Both are synthetic corticosteroids sharing similar pharmacological properties such as effectiveness, adverse side effects, dosing schedules, and drug interactions. Prednisone is a biologically inactive compound which must be converted by liver enzymes to prednisolone before it can act. Therefore, it is prudent to use prednisolone that do not require enzymatic activation in clinical settings in which liver enzymatic activity is impaired (such as severe hepatic failure) (21).

In 1970, Warmolts et al. (22) reported the beneficial effect of alternate-day prednisone in a patient with MG. In the 1970s and 1980s, many clinicians preferred to start prednisone at a low dose (10–25 mg) gradually increasing to 60–100 mg on alternate days, maintain the dose until maximum improvement is reached, and then taper the dose (“dose escalation and de-escalation”). Pascuzzi et al. (23) retrospectively analyzed 116 MG patients treated with prednisone 60–80 mg daily until the onset of improvement, followed by lower-dose alternate-day therapy. They reported that sustained improvement was achieved after a mean of 13.2 days (range, 12 h to 60 days; SD, 11.5 days) of high-dose oral prednisone, and that the duration of high-dose oral prednisone to the time of maximal improvement ranged from 2 weeks to 6 years (mean, 9.4 months; SD, 8.8 months). Finally, they found 80.2% of the patients achieved either remission (27.6%) or marked improvement (52.6%). Sghirlanzoni et al. (24) evaluated the effects of oral corticosteroids in 60 MG patients by long-term observation, and noted improvement in 72% of the patients. In addition, they found the best results in those whose symptoms started after the age of 40 years, and a correlation between the starting dose of prednisone and the rate of improvement. On the other hand, Bae et al. (25) reported that a high daily dosage of prednisone relative to body weight was neither a predictor of exacerbation nor a predictor of early improvement in bivariate correlation analysis. They noted the possibility of steroid-induced exacerbation when prescribing prednisone for MG, especially when treating elderly patients and patients with bulbar dominant or severe disease. Although there are few randomized trials of oral corticosteroids alone, a Cochrane systematic review on corticosteroids for MG published in 2005 concluded that limited evidence from randomized controlled trials does not show any difference in efficacy between corticosteroids and either azathioprine or intravenous immunoglobulin (26).

Dose escalation and de-escalation was also performed traditionally in Japan. Oral steroids were often given using a dose escalation schedule until the symptoms improved sufficiently or until a maximum dose of 50–60 mg/day was reached. Treatment was continued at the highest dose followed by gradual tapering, although the oral steroids usually had to be given chronically with significant risk of adverse events. To address the difficulty of achieving complete remission in adult-onset generalized MG cases, the Japanese clinical guidelines for MG published in 2014 recommend that treatment strategies should aim to maintain health-related quality of life and mental health, considering the possibility of prolonged treatment (27). The guidelines also recommend to reconsider the use of high-dose steroids with escalation and de-escalation, in view of the problems associated with long-term use and the availability of other treatment options.

## Dose-Dependent Effects of Corticosteroids

The expected pharmacologic actions of corticosteroids for treating MG may be divided into an anti-inflammatory action and an immunosuppressive action. Corticosteroids target the postsynaptic membrane to suppress inflammatory reactions including complement-mediated reactions at the endplates. The corticosteroids also inhibit the immune system at multiple sites, including sequestration and decrease of lymphoid cells (28). The anti-inflammatory and immunosuppressive actions of corticosteroids are inextricably linked, perhaps because they both involve inhibition of leukocyte functions (29).

In pharmacokinetics, glucocorticoids (GC), a class of corticosteroids, diffuse across cell membrane and bind to cytoplasmic GC receptor (GR). This binding leads to dissociation of heat shock protein 90, and induces transport of the GC-GR complex across nuclear membrane to the nucleus. In the nucleus, the GC-GR complex binds with various genetic promoters and enhancers of genomic DNA according to the GC responsive elements to regulate the transcription of the target genes (21). These mechanisms would suggest that higher doses of corticosteroids are effective to activate more GRs to obtain favorable anti-inflammatory/immunosuppressive effects. Indeed, it is known that high doses of GCs inhibit immunoglobulin synthesis, kill B cells (30), and decrease production of components of the complement system (31).

Then, the clinical question is: Does higher doses of corticosteroids ensure better outcome in MG treatment?

## Is High-Dose Corticosteroid Superior to Low-Dose in MG Treatment?

### Oral Corticosteroid Therapy and Present Disease Status in MG

As described in the history of MG therapy, oral corticosteroids are traditionally used at high doses with escalation and de-escalation schedules. High-dose oral steroids may not always provide sufficient improvement and may induce long-term steroid-related side effects that impair the quality of life (QOL) of many patients (5, 23).

**Figure 1:**
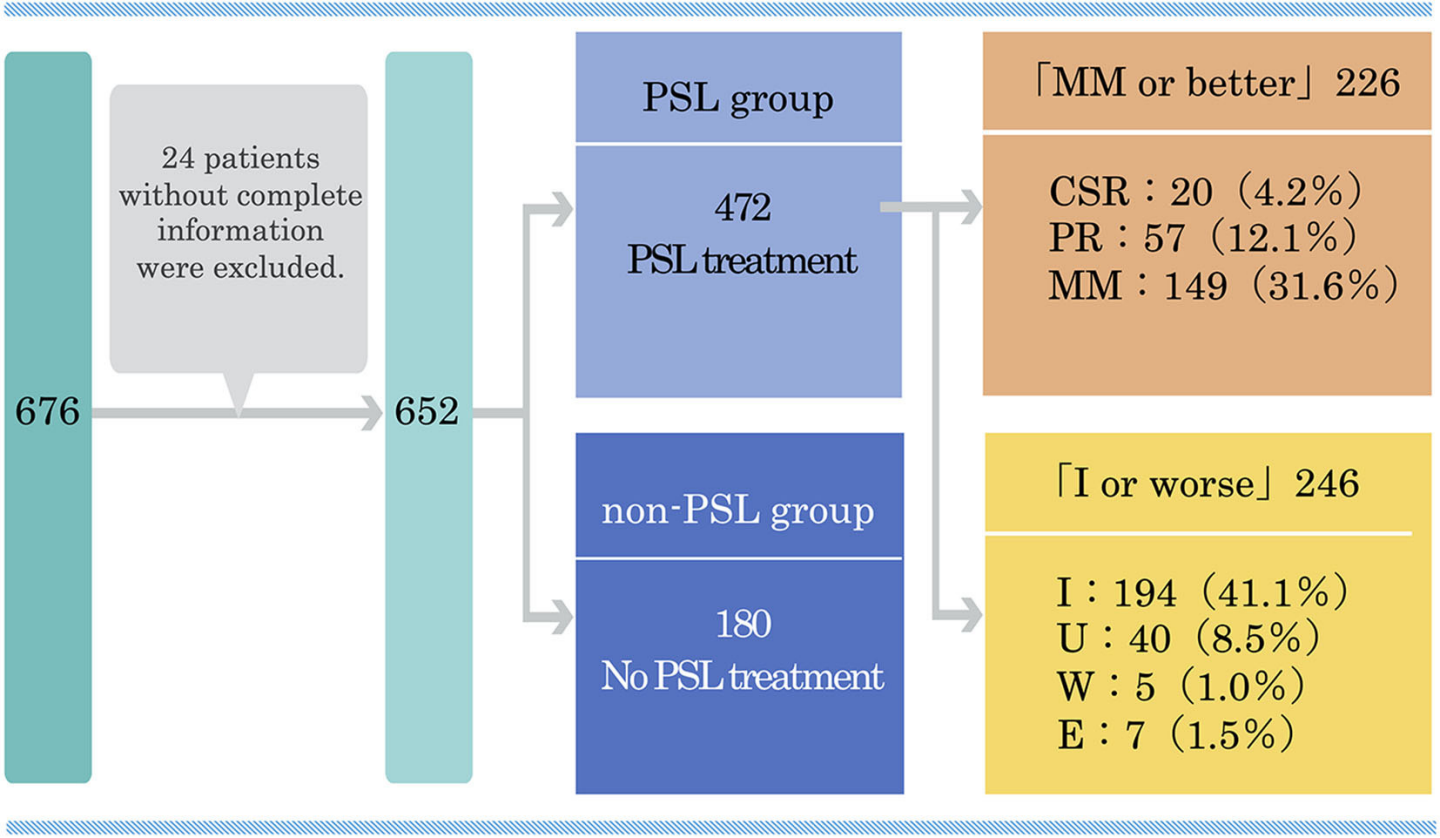
Classification of 472 MG patients treated with prednisolone according to the present disease status in a multicenter, cross-sectional study in 2015. MG, myasthenia gravis; PSL, prednisolone; CSR, complete stable remission; PR, pharmacological remission; MM, minimal manifestations; I, improved; U, unchanged; W, worse; E, exacerbation. This figure is drawn from data published in (32).

We studied 472 MG patients in 2015 to investigate the relationship between oral prednisolone (PSL) dosage and the status of disease at the time of study (current status) (32). These patients were divided by current status into a group of MM or better (complete stable remission, pharmacological remission, MM) (*n* = 226) and a group of improved or worsening status (improvement, unchanged, worse, or exacerbation) (*n* = 246) (Figure 1). There was no significant difference in baseline severity based on clinical classification of MGFA between the MM or better group and the improved or worse group by Pearson χ^2^ test. The treatment duration with PSL was also similar in the two groups (6.5 ± 6.4 vs. 7.1 ± 7.0 years, *p* = 0.56). Patients taking <5 mg/day of oral PSL were more likely to be classified in the MM or better than in the improved or worse group (75.2 vs. 48.8%, *p* < 0.0001). The daily dose of PSL was significantly lower in the MM or better group than in the improved or worse group (4.7 ± 5.3 vs. 7.3 ± 6.5 mg, *p* < 0.0001). The duration of taking PSL ≥10 mg/day was significantly shorter in the MM or better group than in the improved or worse group (10–20 mg/day: 1.9 ± 4.0 vs. 2.1 ± 3.9 years, *p* = 0.01; 20 mg/day or more: 0.6 ± 1.2 vs. 1.4 ± 3.5 years, *p* = 0.0002). In addition, cumulative PSL doses received in the past year was smaller in the MM or better group than in the improved or worse group (1705.9 ± 1791.2 vs. 2460.2 ± 2009.8 mg, *p* < 0.0001).

### Independent Predictors for MM or Better Status From Multivariate Logistic Regression Modeling

Multivariate logistic regression identified MM or better status at peak dose of PSL (*p* < 0.0001) and treatment with PE/PP and/or IVIg (*p* = 0.04) as significant independent positive predictors of achieving MM or better status, and total PSL dose in the past year as the only independent negative predictor (*p* = 0.03). OR was the highest for MM or better status at peak dose of PSL (12.25; 95% CI 7.22–21.43), followed by treatment with PE/PP and/or IVIg (1.92; 95% CI 1.03–3.66) and total dose of PSL in the past year (0.17; 95% CI 0.03–0.88) (Table 1). Other significant variables identified in univariate analyses and entered into the logistic regression model, including the worst QMG score, PSL dose and duration, and use of calcineurin inhibitors (CNI), were not significant independent predictors for the achievement of current status of MM or better.

**Table 1:** Positive and negative predictors for MM or better status from multivariate logistic regression modeling.

**Parameters**		**Odds ratio (95% CI)**	***p*-value**
Positive predictors	MM or better at peak dose	12.25 (7.22–21.43)	<0.0001
	PE/PP and/or IVIg	1.92 (1.03–3.66)	0.04
Negative predictor	Total PSL dose during past 1 year	0.17 (0.03–0.88)	0.03

*Modified from (32). MM, minimal manifestations; PSL, prednisolone; PE, plasma exchange; PP, plasmapheresis; IVIg, intravenous immunoglobulin*.

### Changes of Therapeutic Strategy

These findings lead to the conclusion that higher doses of PSL and longer duration of PSL treatment are not associated with improvement of current condition and that response to PSL treatment is independent of baseline disease severity based on MGFA classification. In other words, MG patients do not possess specific clinical factors associated with poor response to oral corticosteroids, but they are composed of patients who respond well and others who response poorly to oral corticosteroids. Our results also suggest the need for fast-acting combination therapies such as PE/PP and/or IVIg to achieve MM or better in patients who respond poorly to oral corticosteroids. PE/PP, which uses filtration to remove pathological antibodies through three to seven repeated plasma exchanges, has been used in patients with crisis or aggravated MG (8–10). In addition, IVIg is more frequently used as a promising alternative to PE/PP during exacerbations of MG (11–13). However, according to our results, even in the absence of a crisis or exacerbation, fast-acting treatment may be recommended to induce MM or better status at peak doses of oral PSL.

Many patients and physicians prefer to taper corticosteroid doses by combining with other immunosuppressive agents to reduce the side effects of long-term monotherapy with high-dose oral corticosteroids, including mood symptoms and cosmetic problems (33–38). We found that in Japan, percentage of CNI use was high in both the MM or better group and the improved or worse group (51.3 vs. 70.7%). CNIs such as cyclosporine and tacrolimus are recognized as potent corticosteroid-sparing agents, especially in patients receiving high-dose oral corticosteroids for extended periods of time (4, 36–46). If the patients in this study had not been taking CNIs, they may have had to take higher doses of corticosteroids.

**Figure 2:**
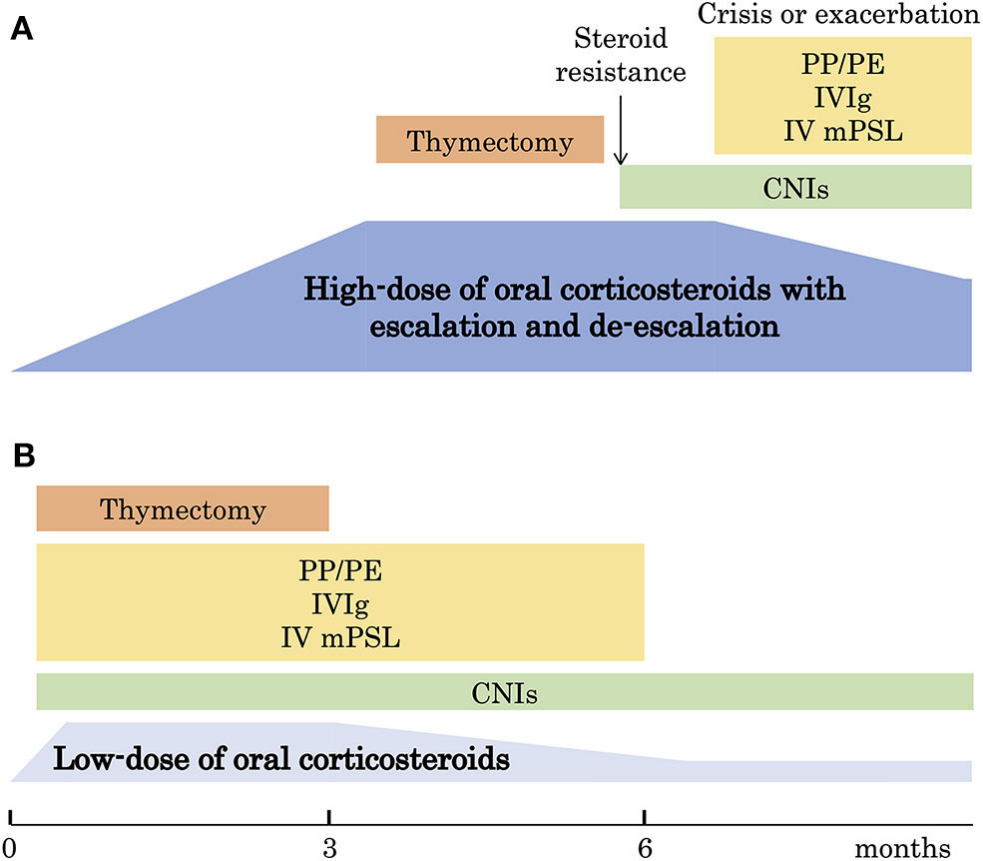
Changes of therapeutic strategy. **(A)** The traditional strategy with high-dose oral corticosteroids with escalation and de-escalation schedule. **(B)** The new strategy with low-dose oral corticosteroids. PE, plasma exchange; PP, plasmapheresis; IVIg, intravenous immunoglobulin; mPSL, methylprednisolone; CNI, calcineurin inhibitor. This figure is drawn from data published in (32).

We proposed a low-dose regimen of oral corticosteroid treatment in MG based on the results of our nationwide survey in 2015 (32) (Figure 2). The low-dose regimen includes low dose of oral corticosteroids, early combination of CNIs, and fast-acting treatments to improve remaining symptoms quickly. The next clinical question is: Is the low-dose regimen superior to the high-dose regimen for long-term prognosis of MG?

## Favorable Regimen of Corticosteroids for MG Treatment

### Oral Corticosteroid Dosing Regimen and Long-Term Outcome in MG

**Figure 3:**
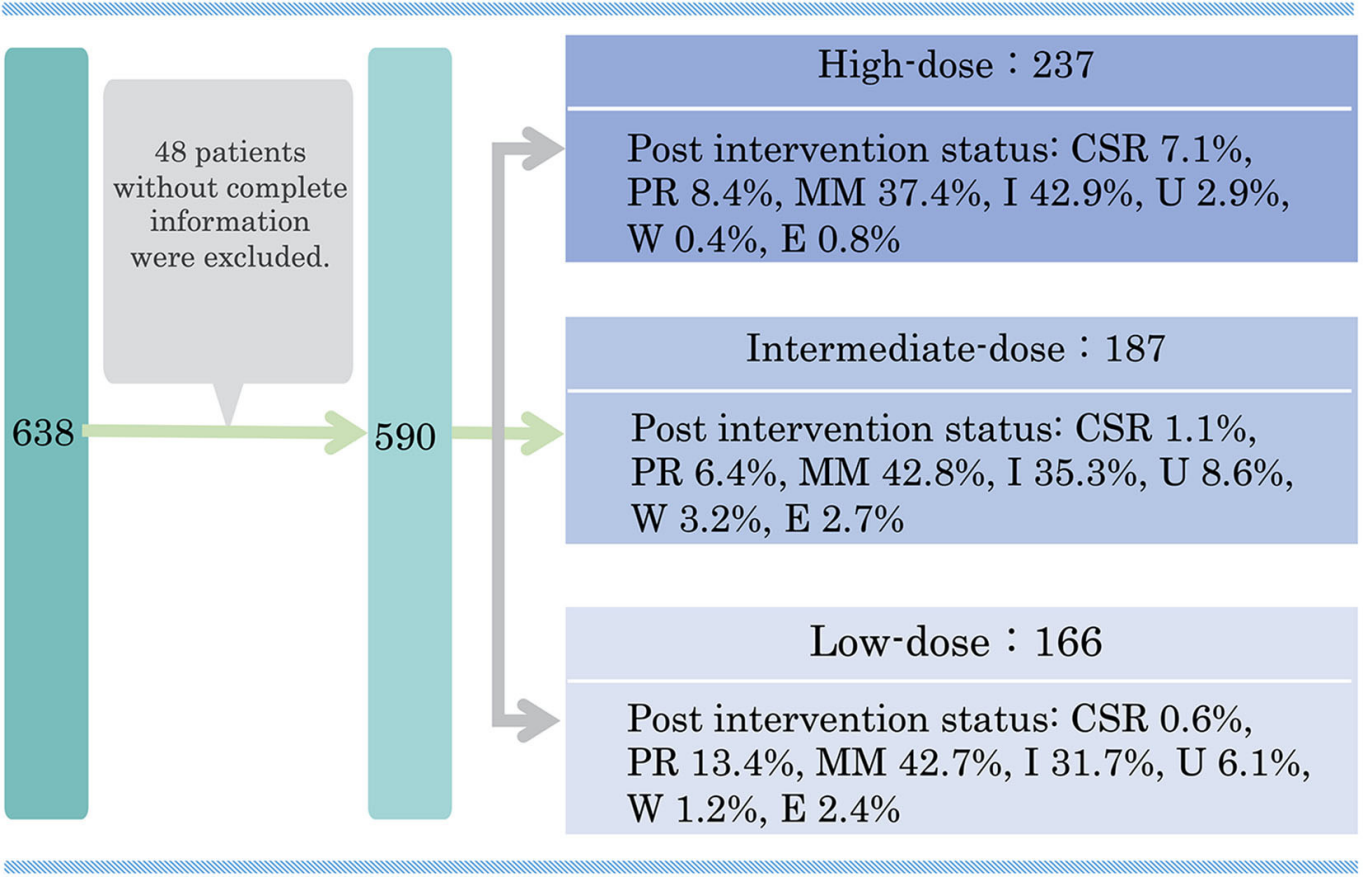
Classification of 590 prednisolone-treated generalized MG patients according to the present disease status in a multicenter, cross-sectional study in 2018. MG, myasthenia gravis; PSL, prednisolone; CSR, complete stable remission; PR, pharmacological remission; MM, minimal manifestations; I, improved; U, unchanged; W, worse; E, exacerbation. This figure is drawn from data published in (47).

Even the international consensus guidance does not include an internationally accepted standard dosing regimen for oral corticosteroids (14). We conducted a multicenter cross-sectional study to examine the correlation between oral PSL administration method and actual achievement of treatment goals (47). A total of 590 patients with generalized MG were classified into three groups according to the dose level of oral PSL during the treatment period: high dose (*n* = 237), intermediate dose (*n* = 187), and low dose (*n* = 166) (Figure 3). Clinical characteristics, history of non-PSL treatment, and prognosis were compared among the three groups. The effect of oral PSL regimen on the achievement of treatment goals was followed over a 3-year treatment period.

### Independent Predictors for MM-or-Better-5 mg Identified by Multivariate Logistic Regression Modeling

Our group also suggests that MM status or better with PSL 5 mg/day or less (MM-or-better-5 mg) is a more realistic treatment goal than CSR, and is achievable by more patients (48).

Multivariate logistic regression analysis identified low-dose regimen, early combination with fast-acting treatment (high-dose methylprednisolone or PE/PP or IVIg), and early use of CNI as predictors of achieving the treatment goal of MM-or-better-5 mg over 6 months (47). ORs for low-dose (vs. high-dose) regimen were 10.4 (*p* < 0.0001) after 1 year, 2.75 (*p* = 0.007) after 2 years, and 1.86 (*p* = 0.15) after 3 years of treatment. ORs for early combination of high-dose methylprednisolone or PE/PP or IVIg were 2.19 at 2 years (*p* = 0.02) and 2.11 at 3 years (*p* = 0.04), and ORs for CNI were 2.09 at 2 years (*p* = 0.03) and 2.36 at 3 years (*p* = 0.02) (Table 2). These results suggest that early combination of low-dose PSL regimens with other therapies is useful for early achievement of treatment goals in patients with generalized MG. However, only 64.1% of patients who received low-dose PSL therapy were able to achieve the treatment goal until 3 years (Table 3). Approximately 35% of patients did not achieve satisfactory outcomes with the new treatment strategy. These results suggest the limitations of current oral corticosteroid therapy and the need to improve the safety and efficacy of corticosteroid therapy.

**Table 2:** Independent predictors of MM-or-better-5 mg for ≥6 months identified by multivariate logistic modeling.

**Parameter**	**Odds ratio (95% CI)**, ***p*****-value**		
	**1 year**	**2 years**	**3 years**
Low-dose regimen (vs. high-dose regimen)	10.4 (4.54–25.2), <0.0001^*^	2.75 (1.31–5.88), 0.007^*^	1.86 (0.79–4.49), 0.15
Early HMP/PP/IVIg	2.04 (0.89–4.78), 0.09	2.19 (1.11–4.42), 0.02^*^	2.11 (1.03–4.44), 0.04^*^
Early use of CNIs	1.59 (0.78–3.24), 0.20	2.09 (1.09–4.06), 0.03^*^	2.36 (1.13–5.09), 0.02^*^

*Modified from (47)*.

*HMP, high-dose intravenous methylprednisolone; PE, plasma exchange; PP, plasmapheresis; IVIg, intravenous immunoglobulin; CNI, calcineurin inhibitor*.

**Table 3:** Achievement of MM-or-better-5 mg for ≥6 months classified by oral PSL dosing regimen.

**Duration**	**High-dose regimen (*n* = 237)**	**Low-dose regimen (*n* = 166)**
1 year	9.6%	52.1%^*^
2 years	29.9%	61.2%^*^
3 years	44.1%	64.1%^*^

*Compiled from data published in (47)*.

* *p < 0.0001 using ANOVA followed by Tukey–Kramer test*.

## Future Considerations

Oral corticosteroids may be effective for good responders regardless of dosage. MG patients who respond well for various reasons may be able to reduce the dosage of steroids with less difficulty because dose reduction may follow the achievement of good outcome but not cause the outcome. Moreover, it is not necessary to use high dosage of oral corticosteroids because a number of new treatment options are now available to achieve good outcome. It is time to reconsider high-dose steroid treatment for MG and seek a novel strategy based on patients' QOL. On the other hand, fast-acting treatment for generalized MG is not suitable for all patients from different countries, especially for patients in developing countries. In this case, further development of steroid drugs is required.

Over the past few decades, considerable efforts have been devoted to increase the potency of corticosteroids while minimizing their side effects by modifying the chemical structure of natural GCs (49). Alternative splicing, alternative translation initiation of mature mRNAs, and post-translational modifications have generated multiple GR isoforms with unique expression, gene regulation, and functional profiles, which have advanced our understanding of the molecular basis of GC susceptibility diversity. Genome-wide GR recruitment studies have shown significant difference of tissue-specific chromatin landscape in GC susceptibility (50).

An important challenge in the clinical application of GC is the heterogeneity of GC response between individuals. Advances in our understanding of GC expression patterns may reveal important mechanisms of poor response in MG treatment. The breakthrough may accelerate not only the design of novel therapeutic strategies for poor responders but also the prediction of enhanced response to corticosteroids for good responders. The understanding of the heterogeneity of GR signaling will permit the development of safer and more effective corticosteroid therapies with improved benefit/risk ratios for MG patients.

## Author Contributions

All authors were involved in conception and design of the work, and in acquisition of data. TI was involved in analysis, interpretation of data, and drafted the article. All other co-authors revised it critically for important intellectual content.

## Conflict of Interest

SS has received personal fees from Alexion Pharmaceuticals, the Japan Blood Products Organization, and Asahi Kasei Medical. YN has received speaker honoraria from Alexion Pharmaceuticals and Japan Blood Products Organization. HM has served as a paid consultant for Alexion Pharmaceuticals, argenx BVBA, and Ra Pharmaceuticals, and has received speaker honoraria from the Japan Blood Products Organization and research support from the Ministry of Health, Labour, and Welfare, Japan. KU has served as a paid consultant for argenx, Ra Pharmaceuticals, and UCB Pharma, and has received speaker honoraria from Alexion Pharmaceuticals. The remaining authors declare that the research was conducted in the absence of any commercial or financial relationships that could be construed as a potential conflict of interest.
